# Community Interventions for Addressing Postpartum Depression Across the United States: A Narrative Review

**DOI:** 10.7759/cureus.106792

**Published:** 2026-04-10

**Authors:** Adedolapo O Ojo, Aniqa Aftabi, Venkata Madhavi Latha Telagarapu, Jonathan Raghubir, Jerusha Gudapati, Adebisi Akindele, Noha Elzaki, Yating Lee

**Affiliations:** 1 Family Medicine, Episcopal Health Services (St John’s Episcopal Hospital), New York, USA; 2 Family Medicine, Touro College of Osteopathic Medicine, New York, USA; 3 Miami Clinical and Translational Science Institute, University of Miami Miller School of Medicine, Miami, USA; 4 Family Medicine, Episcopal Health Services (St John’s Episcopal Hospital), Queens, USA

**Keywords:** community interventions, home visiting program, maternal mental health, postpartum depression, postpartum maternal mental health, postpartum support, social determinants of health, social factors

## Abstract

Postpartum depression (PPD) is a common yet underdiagnosed mental health condition affecting a substantial proportion of new mothers in the United States. Its consequences can be severe, impacting maternal well-being, infant development, and family dynamics. PPD is particularly prevalent in underserved communities, where socioeconomic hardship, limited access to healthcare, cultural stigma, and systemic inequities intensify the risk. Addressing these disparities requires interventions that move beyond traditional clinical approaches and focus on the social, emotional, and environmental determinants of maternal mental health. This narrative review explores a wide range of community-based interventions developed and implemented in the United States to support women experiencing or at risk for postpartum depression. These interventions include nurse-led home visits, telehealth platforms, digital cognitive-behavioral therapy programs, peer-support networks, and culturally grounded co-parenting strategies. Other approaches, such as the use of ergonomic infant carriers and increased exposure to green spaces, have also emerged as innovative means of enhancing maternal-infant bonding and reducing depressive symptoms. Each intervention targets different aspects of the postpartum experience, from improving health literacy and social support to reducing intimate partner violence and enhancing accessibility to mental health resources. By synthesizing findings from diverse settings and populations, this review provides family physicians and community health practitioners with evidence-based tools for identifying, supporting, and treating mothers affected by PPD. Integrating these interventions into primary care and community settings can help mitigate health disparities, strengthen maternal-child relationships, and promote long-term wellness. A focus on culturally sensitive, flexible, and accessible models of care is essential for expanding the reach and impact of PPD prevention and treatment efforts across vulnerable populations.

## Introduction and background

Postpartum depression (PPD) is a mood disorder affecting an estimated 13-20% of new mothers in the United States [1,2]. The International Classification of Diseases 11th Revision (ICD‑11) defines PPD as depressive episodes arising within six weeks of delivery, characterized by persistent low mood, anhedonia, fatigue, sleep and appetite disturbances, impaired concentration, excessive guilt, and, in severe cases, suicidality [3]. Risk is particularly elevated among women over 35 years of age, who experience PPD at nearly twice the rate of younger mothers [4]. Biological contributors, including hypothalamic-pituitary-adrenal axis dysregulation, inflammatory activity, and genetic susceptibility, interact with the hormonal and physiological shifts of the peripartum period, and studies have demonstrated correlations between Edinburgh Postnatal Depression Scale (EPDS) scores and levels of reproductive hormones, thyroid function markers, inflammatory biomarkers, vitamin D, and coagulation parameters [4,5].

When left untreated, PPD can lead to maternal suicide, child harm or neglect, impaired child development, disrupted family functioning, difficulty returning to work, and long‑term mental health conditions [6,7]. Despite its prevalence and consequences, PPD remains underdiagnosed and undertreated, particularly among low‑income, minority, and immigrant women [1,8]. Although effective treatments exist, including pharmacologic therapy and cognitive behavioral therapy (CBT), access remains limited for many mothers facing structural and logistical barriers [9,10].

Social determinants of health, such as poverty, housing instability, lack of paid family leave, low health literacy, and limited access to culturally competent mental health services, contribute significantly to the onset and persistence of PPD [2,10,11]. Women in underserved communities face additional barriers, including transportation challenges, stigma, language obstacles, childcare demands, and limited provider availability [12,13]. Traditional clinic‑based approaches require mothers to navigate these same barriers, perpetuating inequities in diagnosis and treatment.

Community‑based interventions offer a promising alternative by bringing support directly into the environments where mothers live, parent, and seek informal help. Through home visiting programs, digital platforms, peer networks, community health workers, and culturally grounded programming, these models reduce logistical barriers, enhance trust, and align care with the lived realities of underserved families [8,13,14]. Digital tools, in particular, have shown potential to expand access and reduce symptoms through CBT‑based modules, psychoeducation, and peer support [14-17].

To address these gaps and provide clarity for clinicians and community partners, this narrative review synthesizes evidence on community‑based interventions implemented in the United States to prevent or reduce PPD, with a particular focus on underserved populations. Specifically, the review examines five categories of non‑traditional, community‑anchored approaches: (i) nurse home visiting programs, (ii) digital and telehealth‑based mental health supports, (iii) culturally tailored coparenting and social support interventions, (iv) practical tools that promote bonding and maternal well‑being, and (v) nature‑based strategies that enhance stress regulation and emotional health. The objective is to evaluate the effectiveness, strengths, and limitations of these interventions and to generate actionable insights that can guide family physicians, primary care clinicians, and community organizations in integrating these resources into routine postpartum care.

Methodology

Search Strategy

We conducted a targeted search of PubMed and reviewed additional secondary references identified through citation tracking. The search included both full‑text and free‑text articles. Keywords used were “community interventions”, “postpartum depression”, “maternal mental health”, and “home visiting”. This was a narrative review, and thus, the search strategy was designed to identify conceptually relevant studies rather than to produce an exhaustive or reproducible systematic search. Reference lists of included articles were also screened to capture additional eligible studies.

Study Selection Criteria and Process

Studies were included if they were published in English between 2000 and 2024, were randomized controlled trials (RCTs), pilot studies, retrospective studies, or observational studies, examined community‑based interventions delivered in the United States, and reported outcomes related to PPD symptoms or scores, including both improvements and null findings. We excluded systematic reviews, meta‑analyses, and other literature review articles, as well as studies focused solely on pharmacologic treatments or conducted outside the United States.

Titles and abstracts were screened for relevance, followed by full‑text review. Studies were selected based on conceptual alignment with the review objective: identifying community‑based, non‑pharmacologic interventions that address postpartum depression within a biosocial framework.

Data Extraction and Synthesis 

For each included study, we extracted information on study design and sample characteristics, intervention components, delivery model, and duration, primary and secondary outcomes, measures of postpartum depression (e.g., Edinburgh Postnatal Depression Scale (EPDS), Patient Health Questionnaire-9 (PHQ-9), Hamilton Rating Scale for Depression (HRSD)), implementation features such as feasibility, engagement, cultural tailoring, and acceptability, and reported limitations and contextual factors. 

A thematic synthesis approach was used to organize studies into five categories: (i) Nurse home‑visiting and in‑person support, (ii) Digital and telehealth interventions, (iii) Culturally tailored coparenting and social‑support programs, (iv) Practical bonding tools, and (v) Nature‑based interventions. Themes were developed iteratively by comparing intervention mechanisms, target populations, and outcome patterns. Findings were interpreted within a biosocial model, emphasizing how biological, behavioral, relational, and structural factors interact to shape maternal mental health.

*Quality and Rigor Considerations* 

Although formal risk‑of‑bias scoring was not conducted, consistent with narrative review methodology, we considered: sample size and representativeness, study design (RCT, quasi‑experimental, pilot), measurement validity and reliability, attrition and engagement patterns, and generalizability to underserved populations. These factors informed the interpretation of findings and the identification of interventions with the strongest evidence and greatest real‑world promise.

## Review

To assess how community-based strategies can mitigate PPD, the available literature was categorized into five key thematic categories as mentioned. Each category addresses distinct barriers to care or stressors affecting women with PPD. 

Nurse home visits and in-person support

Home visitation programs aim to reduce maternal stress, enhance parenting capacity, and improve mental health outcomes by bringing support directly into the home environment. These interventions are especially impactful for rural, low-income, or socially isolated women. Low health literacy, combined with limited access to healthcare, is a common barrier for high-risk, low-income, rural women, which can impact maternal and infant health outcomes.

A study by Mobley et al. evaluated the impact of home visits by registered nurse case managers (RNCMs) on improving health literacy and mental health among low-income, high-risk African American and White women in rural Georgia [18]. In their study, 106 women participated in the Enterprise Community Healthy Start (ECHS) program, which provided them with RNCM home visits from the prenatal period through 24 months postpartum: beginning from two months after admission, at two months postpartum, and every six months thereafter. These RNCMs offered education on health, parenting, and how to effectively use healthcare services, aiming to improve maternal health literacy. RNCMs administered life skills progression (LSP) assessments to measure maternal healthcare literacy (M-HCL) and maternal self-care literacy (M-SCL). Among the 106 women, baseline scores were inadequate (<4), and initially, 83% of participants showed inadequate M-HCL, and 30% showed inadequate M-SCL. However, after repeated visits, significant improvements were observed in both literacy scores from prenatal to first postpartum (p < .01) and final postpartum assessments (p < .01). Gain scores showed progression in both LSP/M-HCL (p < .0001) and LSP/M-SCL (p < .0001) [18].

In the same study by Mobley et al., depression screenings at various stages showed that women with lower health literacy scores tended to have higher depression scores [18]. RNCM notes indicated that social challenges, such as lack of support from the baby’s father and relationship conflicts, contributed to higher depression levels in these women. Depressive symptoms were measured during pregnancy and postpartum using the Beck Depression Inventory (BDI) and the EPDS. EPDS scores did not show a statistically significant reduction following delivery in either the combined intervention groups (p = .069) or Group IV (p = .32), indicating no significant improvement in PPD symptoms. However, the PPD scores did not explain differences in intervention outcomes, suggesting that the observed effects were independent of PPD symptom levels [18].

The RNCM health literacy intervention primarily focused on improving maternal healthcare and self‑care literacy among high‑risk rural women through longitudinal, education‑centered home visits [18]. Its major strength was the substantial, statistically significant improvement in maternal health literacy across the pregnancy and postpartum periods, demonstrating that structured nurse‑delivered education can effectively enhance women’s capacity to navigate healthcare systems and engage in self‑care. However, the intervention’s key limitation was its lack of impact on PPD symptoms; neither EPDS nor BDI scores showed significant change over time. This suggests that while health literacy is an important determinant of maternal well‑being, educational support alone is insufficient to influence the complex psychosocial and structural factors that drive PPD. The findings indicate that RNCM programs may be most effective when integrated with targeted mental‑health or psychosocial components that address relationship stress, social support deficits, and other contextual contributors to maternal mood.

An RCT by Dodge et al. evaluated a nurse-led community home visitation program designed to address a broad range of maternal and infant health challenges in vulnerable families [19]. This study emphasized a biosocial and health model, recognizing that PPD emerges from the interaction of individual vulnerabilities, social stressors, and the resources available within women’s natural environments. Families were randomly assigned to the Family Connects Program or treatment-as-usual. Nurses made regular home visits to assess needs across 12 domains (including parent/infant health, medical home, child care planning, maternal depression/anxiety, and social support) and provided tailored support through brief interventions, community resource connections, or crisis services. These included substance abuse treatment programs, parenting support services, housing assistance, and links to peer support groups. The approach emphasized early, individualized intervention, with nurses tailoring their visits to address each family's unique circumstances.

Dodge et al.'s analysis included 158 intervention families and 158 controls [19]. Nurses addressed minor problems in 52% of families and linked 42% to community resources. Child abuse investigations averaged 0.10 (0.30) in the intervention group vs. 0.18 (0.56) in controls (b = -0.09; 90% CI, -0.12 to -0.01; 95%CI, -0.18 to 0.01; P = .07). Maternal anxiety/depression rates were lower in the intervention group (18.2%) compared to controls (25.9%) (b = -7.70; 90%CI, -15.2 to -0.1; 95%CI, -16.6 to 1.3; P = .09). The program demonstrated strong outcomes in maternal mental health and child well- being. Participation rates were high, and families receiving the intervention reported reduced levels of maternal anxiety and depression. The program was also associated with fewer child maltreatment investigations and stronger community connections [19].

A key factor in the program’s success was its ability to build trust and deliver focused, professional support within the family’s home environment, enhancing access to care while reducing barriers such as stigma and transportation. Importantly, the study also emphasized the value of continuous quality monitoring and evaluation. Maintaining program fidelity and ensuring interventions remain responsive to evolving family needs were seen as essential to sustaining long-term effectiveness. The authors concluded that community-based nurse home-visiting programs can be highly successful when structured around tailored support, culturally competent care, and strong community partnerships [19]. Scaling such programs with attention to quality control and equitable access offers a promising strategy for improving maternal mental health and reducing disparities in early childhood outcomes.

These findings highlight the effectiveness of home-visiting models that not only offer medical or psychological support but also address the social determinants of health, such as housing instability, substance use, and limited social support, that often underlie poor maternal-child outcomes. Community‑based interventions are therefore critical, as they operate within the ecological contexts that shape maternal mental health [20]. Community agency-delivered nurse home visitation increases service connections and reduces maternal anxiety/depression rates, with modest effects on child abuse investigations. A limitation of the Family Connects Program is that implementation requires a trained nursing workforce and strong community partnerships [19]. Nonetheless, the interpretation is clear: Family Connects is an effective home‑visiting model for maternal mental health because it directly targets the multilevel drivers of PPD through individualized, context‑responsive care. Its comprehensive design positions it to influence both maternal well‑being and child safety in ways that education‑focused or advocacy‑focused models cannot.

Another study explored the impact of incorporating community doulas into home-visiting programs for young, low-income mothers across four Illinois communities [21]. In this study, Doula home-visiting services increased maternal engagement in childbirth-preparation classes but did not affect birth or postpartum outcomes. A doula acts as a mother's advocate by listening to her decisions made during the delivery process with empathy and knowledge [22]. Decisions that are made with the mother and her child's best interests in mind are empowered by the doula. Positive experiences are routinely reported in studies that look at the relationship between delivering women and their doulas [23].

The study targeted a racially diverse population with a mean participant age of 18.4 years, reflecting a high-need demographic often at elevated risk for adverse maternal and infant outcomes [21]. Participants in the intervention group received personalized, culturally attuned support from trained community doulas throughout pregnancy and the postpartum period. These doulas provided guidance on childbirth preparation, breastfeeding, infant care, and maternal well-being through consistent home visits tailored to each mother's needs. The control group received standard case management services, allowing for a comparison of outcomes with and without doula involvement. In a sample of 312 pregnant women (mean age 18.4 years) from four high-poverty Illinois communities, mothers in the intervention group were seen to be significantly more likely to attend childbirth preparation classes, and no significant differences were observed in Caesarean delivery, birthweight, prematurity, or PPD. Additionally, they were less likely to use pain medication during labor, suggesting increased confidence and coping [21]. This limitation suggests that while doulas effectively promote maternal engagement and positive parenting behaviors, the model does not directly address the psychological or structural determinants of PPD. The interpretation is that doula programs offer valuable relational and cultural support, particularly for marginalized mothers, but may require integration with mental health or social‑determinant interventions to influence maternal mood outcomes.

Community-based interventions delivered by visiting nurses and doulas aim to improve maternal health literacy and reduce PPD, thereby strengthening the mother-infant relationship [19,21]. However, implementation may be challenging among high-risk women from certain racial or cultural backgrounds, where cultural beliefs or family dynamics may limit acceptance of outside support [24]. Building trust through sustained community engagement and culturally sensitive outreach is essential to increasing participation and openness to care [25]. Common stressors such as partner conflict and limited paternal involvement, both strongly associated with worse maternal mental health outcomes, cannot be fully eliminated [26]. Nevertheless, gradual inclusion of family members in health literacy programs may improve maternal depression outcomes, though engaging the entire family remains a significant challenge [26,27]. Additional barriers include the need for ongoing monitoring and timely adaptation of interventions to prevent worsening outcomes [28]. Effective implementation may also require flexible budgeting and, in some cases, financial assistance tailored to the specific needs of high-risk populations based on prior community experience [29].

An RCT evaluated a relationship‑focused behavioral coaching program designed to enhance maternal-infant interactions among mothers experiencingPPD [30].The study followed 134 postpartum mother-infant dyads over a nine-month period, with 125 completing the protocol. Participants were recruited from two major medical centers in southeastern New England and screened for PPD at six weeks postpartum. Eligible mothers were randomized into treatment or control groups. The treatment group received nurse-led home visits from six weeks to nine months postpartum, focused on enhancing maternal-infant relational effectiveness, called Communicating and Relating Effectively (CARE) for the treatment group. The CARE intervention was designed to improve maternal-infant interaction quality among mothers with PPD through structured, nurse‑led behavioral coaching from six weeks to nine months postpartum. Nurses coached depressed mothers to improve interactions with their infants by targeting specific behaviors and encouraging positive responses. Both groups had their interactions video-recorded at four intervals: six weeks, three months, six months, and nine months postpartum. In the final phase, qualitative insights were gathered via focus groups and individual interviews. Both intervention and control groups showed improvements in maternal-infant interaction and depression severity, with no significant group difference. Thus, the specific efficacy of the CARE intervention was only partially supported. Researchers noted that nurse involvement with both groups, including empathic listening, instructions during video sessions, and support with referrals, may have unintentionally acted as a treatment for the control group [30]. These findings suggest that even general nurse-led home visits, beyond the structured CARE protocol, may offer meaningful mental health and bonding benefits for mothers with PPD. This underscores the need for clearer differentiation between intervention and control conditions and suggests that relational coaching may require integration with more intensive mental health strategies to produce measurable reductions in PPD.

Across the four home‑visiting interventions reviewed, the Family Connects program demonstrated the strongest evidence for improving maternal mental‑health outcomes [19], whereas the RNCM health‑literacy model and the community doula program improved proximal outcomes such as literacy, parenting behaviors, and maternal engagement, but did not reduce PPD [18]. The Family Connects model produced a meaningful reduction in maternal anxiety/depression and fewer child maltreatment investigations. The CARE relational‑coaching trial similarly did not outperform general nurse contact, suggesting that nonspecific nurse support may itself be beneficial but insufficient to produce differential effects [30]. Family Connects appears most effective because it directly addresses the biosocial determinants of PPD through comprehensive assessment, tailored support, and active linkage to community resources. This multi‑domain, community‑embedded approach is better positioned than education‑focused or advocacy‑focused models to influence maternal mental health trajectories.

Digital and telehealth interventions

Digital and telehealth-based interventions offer scalable, flexible models to deliver mental health support, particularly for women facing transportation, time, or childcare barriers. These tools often rely on CBT, peer support, or mindfulness-based approaches.

A study evaluated an eight‑week mindfulness‑based videoconference intervention for pregnant and postpartum women experiencing or at risk for PPD [31]. This group videoconference session integrating mindfulness-based cognitive therapy (MBCT) showed promising trajectories in reducing perinatal depressive symptoms. Conducted in both urban and rural clinical settings, the study involved weekly one-hour sessions led by trained mental health professionals and based on the UPLIFT (Using Practice and Learning to Increase Favorable Thoughts) program, which integrates MBCT. Participants received preliminary training to ensure comfort with the telehealth platform. A total of 47 women participated, with 51% having mild to moderate depressive symptoms and 49% considered high-risk but asymptomatic. Strong attendance and high survey completion rates were reported. EPDS scores in the symptomatic group decreased significantly from 13.5 at baseline to 10.3 at two months post intervention. The high-risk group maintained stable, low EPDS scores throughout, suggesting possible prevention of symptom onset [31].

The strengths of the study included high feasibility, strong attendance, and significant reductions in depressive symptoms among symptomatic participants [31]. The intervention also appeared to prevent symptom onset among high‑risk but asymptomatic women, suggesting both treatment and preventive potential. However, limitations included a small sample size, limited racial and socioeconomic diversity, and the mixing of pregnant and postpartum participants, which complicates the interpretation of differential effects. The findings indicate that synchronous, facilitator‑led telehealth groups can effectively deliver MBCT and reduce depressive symptoms, but larger, more diverse trials are needed to confirm generalizability and clarify which subgroups benefit most.

Another study evaluated the effectiveness and user experience of the Mothers and Babies Online Course (eMB), a web-based CBT- informed intervention aimed at preventing and managing PPD [32], adapted from an established in‑person program. The eMB program expanded the reach of CBT-based prevention strategies but showed limited engagement beyond initial lessons. Participants were recruited via email, social media, and word of mouth, and upon consent, were asked to complete a baseline survey on mood, perinatal history, maternal mental health knowledge, and demographic data. Lessons were delivered through interactive modules that included readings, videos, worksheets, and reflection questions. Participants could complete the lessons in any order, and each lesson included a rating scale and a free-text feedback option to assess perceived usefulness and understanding [32].

Out of 208 total participants in the study by Barrera et al., 63% were healthcare providers and 37% were perinatal women (pregnant or postpartum) [32]. Among perinatal participants, 66.7% identified as White/European, and 87.7% were United States residents. Perinatal women generally rated their maternal mental health knowledge lower and reported lower baseline mood scores compared to healthcare providers. The primary strength of the eMB program was its scalability and ability to reach geographically dispersed participants, including those facing barriers such as transportation, childcare, or cost. However, despite high initial interest, 22.6% of participants never accessed the course, and among those who did, engagement tapered off quickly; only 4.4% completed all eight lessons. Most participants accessed Lesson 1 first; however, it received the lowest usefulness ratings. Average lesson completion was approximately two lessons per participant. Encouragingly, perinatal women were more engaged with interactive elements than healthcare providers (33.3% vs. 22.9%), and nearly 40% of all users gave feedback on at least one lesson, which indicated that while the program was appreciated and potentially valuable, improvements were needed, particularly in tailoring content to individual needs and improving lesson interactivity and relevance [32]. This signifies that while self‑guided digital CBT platforms can broaden access, their effectiveness is constrained by high attrition and limited interactivity. Without personalization, human support, or engagement‑enhancing features, self‑paced programs may struggle to produce meaningful mental‑health improvements in real‑world settings.

Given that PPD affects a substantial proportion of new mothers and remains a major complication following childbirth [1], the eMB platform was developed to help bridge gaps in access to maternal mental health care, especially for mothers facing socioeconomic and logistical barriers such as transportation, childcare, and cost [12,13,33]

A pilot study was conducted to assess the feasibility, acceptability, and potential effectiveness of MomMoodBooster (MMB), an interactive web-based CBT program supported by regular phone coaching to treat mild to moderate PPD [34]. The guided web-based MMB program demonstrated high engagement and significant reductions in PPD symptoms. The study included 53 women from the United States who were under nine months postpartum and met criteria for mild to moderate depressive symptoms. Participants received access to the MMB platform and were paired with a personal coach who provided regular phone support and helped guide the use of CBT techniques. Over a six-month period, participants completed standardized assessments at baseline, three months, and six months, including the PHQ-9, HRSD, and other tools measuring mood, activity, and parenting self-efficacy. Its strengths included low cost, high engagement, strong user satisfaction, and significant, sustained reductions in depressive symptoms, with 77% of participants achieving clinically meaningful improvement. Participants reported high levels of satisfaction with the program, particularly valuing the structured guidance and regular support from their assigned coach [34]. However, limitations included a quasi‑experimental design without a randomized control group, a small sample size, and a participant pool skewed toward higher‑income, highly educated women, which limits generalizability. The findings suggest that MMB is a promising, low-cost, and accessible digital intervention that can reduce depressive symptoms in postpartum women [34,35]. The addition of a personal coach likely enhanced adherence and therapeutic alliance, distinguishing MMB from self‑guided digital programs.

The interpretation is that guided digital CBT with structured content plus human support shows the strongest and most consistent evidence for reducing PPD among digital interventions. Future large-scale randomized trials in more diverse populations are needed to validate its effectiveness and scalability

A study by Baumel et al. conducted at New York evaluated the integration of a digital peer-support platform into care for women diagnosed with PPD, and 19 mothers with a clinical diagnosis of PPD were enrolled and introduced to the 7Cups platform, which offers 24/7 text-based emotional support from trained volunteers, many with lived experience of perinatal mood disorders [36]. The study aimed to assess the feasibility, user acceptance, and early effectiveness of the 7Cups platform when used alongside standard clinical treatment. Peer support via the 7Cups platform was feasible, acceptable, and associated with reductions in depressive symptoms. Participants were guided on platform use and encouraged to explore peer chat, self-help exercises, and emotional tracking tools. The study employed a non-randomized design and used propensity score matching to compare outcomes with a treatment-as-usual (TAU) control group. Key outcomes included feasibility (recruitment and engagement), acceptability (participant feedback), and preliminary efficacy (change in EPDS scores). Participants engaged with the platform a median of 12 times over two months, totaling 175 minutes of use, primarily during evening and nighttime hours when clinical support is typically unavailable. No safety concerns were reported during interactions with peer supporters. Results showed a significant within-group reduction in EPDS scores for the 7Cups users (p < .001; Cohen’s d = 1.17). Although the difference between the 7Cups and TAU groups was not statistically significant, a moderate effect size favoring the intervention group was observed (p = .05; Cohen’s d = 0.58) [36].

The strengths of the 7Cups intervention included high feasibility, strong acceptability, and meaningful within‑group reductions in EPDS scores, with participants engaging most frequently during evening and nighttime hours when clinical support is typically unavailable [36]. The platform’s ability to extend support beyond traditional care hours is a notable advantage. However, limitations included a small sample size, a non‑randomized design, and reliance on propensity score matching, which restricts causal inference. Although between‑group differences did not reach statistical significance, a moderate effect size favored the intervention. The interpretation is that digital peer support may serve as a valuable adjunct to clinical care, particularly for addressing isolation and providing real‑time emotional support, but larger randomized trials are required to determine its independent impact on PPD.

Across the digital and telehealth interventions reviewed, clear differences emerged in engagement, therapeutic intensity, and mental‑health impact. The mindfulness‑based videoconference program demonstrated strong feasibility and produced meaningful reductions in depressive symptoms among symptomatic women, suggesting that synchronous, facilitator‑led telehealth can effectively deliver MBCT content [31]. In contrast, the self‑guided eMB program expanded reach but suffered from low engagement and minimal lesson completion, limiting its potential to influence PPD [32]. The 7Cups digital peer‑support platform showed promising within‑group reductions in depressive symptoms and offered valuable off‑hours emotional support, yet its non‑randomized design and small sample size constrain conclusions about independent efficacy [36]. Among all digital interventions, MMB demonstrated the most consistent and clinically meaningful improvements in PPD, with 77% of participants achieving significant symptom reduction [34]. Its relative effectiveness likely reflects the combination of structured CBT content with regular human coaching, which enhances adherence, therapeutic alliance, and skill application. Taken together, these findings indicate that guided digital CBT models with integrated human support, exemplified by MMB, currently represent the most effective and scalable digital approach for reducing PPD, while self‑guided or peer‑only platforms may require additional engagement or support components to achieve comparable outcomes.

Integrating structured mental‑health interventions such as CBT into home‑visiting programs may substantially enhance their effectiveness in addressing PPD. The strong symptom reductions observed in guided digital CBT programs like MMB demonstrate the value of structured therapeutic content paired with human support, while the Family Connects home visiting model highlights the importance of tailored, culturally competent care that addresses social determinants of health. One possible explanation for the improvements in maternal anxiety/depression observed in the Family Connects trial is the program’s ability to facilitate timely connections to mental health services, enabling access to evidence‑based treatments such as CBT. Combining these strengths, structured CBT content, personalized coaching, community engagement, and targeted linkage to mental health resources may offer a more comprehensive and scalable strategy for reducing PPD, particularly in underserved populations where both psychosocial stressors and barriers to care are prevalent.

Culturally tailored coparenting and social support

The Focused Coparenting Consultation (FCC) trial evaluated a culturally grounded prenatal intervention designed for unmarried Black and mixed‑race couples expecting their first child. Conducted in a community setting with 138 low‑income couples, the study addressed the unique relational and socioeconomic challenges that shape the transition to parenthood for underserved Black families [37]. Unmarried Black parents are often overlooked in coparenting research [38,39], despite facing elevated stress, limited social support, and structural inequities that influence early parenting dynamics.

FCC consisted of six prenatal sessions led by a male-female mentor team and focused on strengthening communication, collaboration, and problem‑solving between partners. Compared with treatment as usual, mothers receiving FCC reported better coparenting communication and greater father involvement at three months postpartum, and both parents experienced reductions in psychological intimate partner violence. However, depressive symptoms declined similarly in both groups, indicating that while relational interventions can enhance family functioning, they may not independently reduce maternal depression [37].

Although the FCC trial provides important evidence for culturally grounded coparenting support among Black families, it represents only one part of a broader but still emerging landscape of culturally responsive perinatal interventions in the United States. Because our search strategy focused specifically on PPD, several relevant programs were not captured in the primary review; however, they offer valuable context for understanding how culturally tailored approaches function across diverse underserved communities.

For example, a culturally adapted perinatal depression-prevention program delivered through home visiting for low‑income Hispanic and African American mothers demonstrated improvements in maternal mood and parenting outcomes [40]. Similarly, the Supporting Father Involvement program, implemented with racially diverse, low‑income families, strengthened coparenting relationships and reduced conflict, highlighting the importance of relational support in early parenting [41]. Among Indigenous communities, the Family Spirit home‑visiting program for American Indian adolescent mothers has shown significant benefits for maternal mental health, parenting self‑efficacy, and child development [42].

Together, these studies illustrate that culturally responsive, relationship‑focused interventions can benefit a range of underserved populations, even though the evidence base remains uneven across groups. They also reinforce a key insight from the FCC trial: while culturally grounded relational work reliably enhances coparenting dynamics, parental engagement, and family well‑being, its impact on maternal depression is most likely to be realized when paired with targeted mental health components.

Practical tools to promote bonding and reduce depression

This theme explores creative, low-cost strategies designed to strengthen maternal-infant bonding while reducing PPD, particularly in underserved populations. Innovative interventions, such as the use of ergonomic infant carriers, have shown early promise in enhancing physical closeness, which supports both maternal mood and bonding in the critical early postpartum period [43]. These approaches offer scalable and culturally adaptable tools that can be integrated into existing community-based programs, providing accessible mental health support without relying solely on traditional clinical models [44,45]. As mental health challenges in postpartum women are often linked to both emotional and socioeconomic stressors, such interventions may play an important role in broader strategies to improve maternal well-being.

An RCT was conducted to test whether providing an ergonomic infant carrier during pregnancy could reduce PPD symptoms among low-income mothers [43]. A total of 100 women were randomly assigned to receive the carrier either prenatally (intervention group) or at six months postpartum (control group). Providing ergonomic infant carriers during pregnancy increased mother-infant physical contact and was associated with reduced PPD symptoms at six weeks postpartum; mothers in the intervention group had significantly lower EPDS scores compared with those in the control group (β = −0.541, p = .042). The intervention was well received, with 79.5% carrier usage [43]. Practical, low‑cost tools that increase mother-infant physical contact represent an emerging strategy for supporting maternal mental health in underserved communities.

The RCT evaluating prenatal provision of ergonomic infant carriers primarily focused on enhancing early bonding by increasing opportunities for physical closeness during the postpartum period. Its strengths included high acceptability, nearly 80% of mothers used the carrier, and a significant reduction in PPD symptoms at six weeks, with intervention participants demonstrating lower EPDS scores than controls [43]. These findings suggest that simple behavioral tools that facilitate touch, responsiveness, and proximity may positively influence maternal mood through both biological pathways (e.g., oxytocin release, stress regulation) and relational mechanisms that strengthen early caregiving. However, the study’s limitations include a relatively small sample size, a short follow‑up window, and limited demographic diversity, which constrain generalizability and prevent conclusions about long‑term mental health impact. This indicates that while ergonomic carriers show promise as a scalable, culturally adaptable adjunct to postpartum support, particularly in low‑resource settings, future research should examine their effectiveness in larger, more diverse populations and explore how such tools might be integrated with psychosocial or mental health interventions to maximize benefits for maternal well‑being.

Nature-based interventions

A pilot RCT that tested an intervention aimed at increasing time spent in greenspace among urban-dwelling predominantly Black postpartum women from low-resource neighborhoods in Philadelphia increased postpartum women’s time spent outdoors but did not significantly reduce depression scores [46]. Spending time in nature has known mental health benefits, including reducing depression and stress [47]. The intervention combined a brief educational session with personalized support from a Nature Coach, a community health advocate sharing the participants’ background, and digital nudges via text messages to encourage weekly nature visits over four weeks [46]. The Nature Coach engaged participants through home visits, park visits, and phone check-ins, while the digital component provided personalized feedback and reminders to foster engagement. GPS data tracked the frequency and duration of visits to green spaces. Although the study showed no statistically significant differences in PPD scores (likely due to small sample size), participants in the intervention group tended to spend more time and visit nature more frequently than the control group (IRR 2.6, 95%CI 0.96-2.75, p = 0.059). Among women who completed the intervention (13 of 17), nature visits were three times higher than controls (IRR 3.1, 95%CI 1.16-3.14, p = 0.025). No significant differences were found in EPDS scores, likely limited by the small sample size [46].

Nature‑based interventions represent a promising but still emerging approach to supporting postpartum mental health, particularly in underserved urban communities with limited access to restorative environments. The Nurtured in Nature pilot RCT primarily focused on increasing postpartum women’s exposure to green space through a behavioral‑economics-informed model that combined brief education, personalized support from a culturally concordant Nature Coach, and digital nudges encouraging weekly outdoor visits [46]. The intervention’s strengths included high feasibility, strong participant engagement, and meaningful behavioral change: women in the intervention group spent more time in nature and visited green spaces more frequently than controls, with GPS‑verified increases in both duration and frequency of outdoor exposure. These behavioral gains are notable given the well‑established links between nature exposure, stress reduction, and improved emotional well‑being. However, the study’s key limitation was its lack of significant impact on PPD symptoms, likely due to its small sample size, short intervention duration, and variability in the quality, safety, and accessibility of available green spaces. This suggests that while nature‑based interventions can successfully modify health‑promoting behaviors and may contribute to improved well‑being, their direct effects on PPD remain uncertain. Larger, adequately powered trials are needed to determine whether increased nature exposure can translate into measurable mental‑health benefits, and how such interventions might be integrated with psychosocial or clinical supports to enhance their impact in low‑resource, predominantly Black communities.

The latter themes in this review were supported by only one study each, underscoring the limited development of these intervention models in the United States literature. Although these pilot studies demonstrate feasibility and potential benefit, the evidence remains preliminary. Future research should prioritize expanding these underexplored areas to determine their scalability, effectiveness, and relevance for diverse postpartum populations.

The studies are summarized in Table 1. Table 2 categorizes the studies by intervention themes and the contributing factors to PPD that they address.

**Table 1:** Summary of community intervention studies addressing postpartum depression based in the United States RNCM: registered nurse case manager; CARE: centered behavioral coaching program; FCC: Focused Coparenting Consultation; RCT: randomized controlled trial

Study (author, year, location)	Aims and purpose	Method and sample	Intervention	Outcomes and key findings
Mobley et al.(2014), Georgia [19]	To determine the impact of home visits on maternal health literacy progression among low-income, high-risk, rural perinatal African American and White women	Retrospective observational study of 106 women, prenatal period to 24 months post- partum	Home visits were conducted by RNCMs. The first Life Skills Progression assessment was completed at two months after admission to the program and subsequently at two months post- partum and every six months	Home visitation by nurses significantly improved health literacy among rural, high-risk perinatal women leading to improved health outcomes for both mothers and infants, including better birth outcomes and potentially lower infant mortality
Dodge et al. (2019), North Carolina [19]	To identify and evaluate individual family needs, perform brief interventions, and connect families with community-based resources. To evaluate the implementation and impact of the Family Connects (FC) program when administered through a community agency	RCT: 936 births at Duke University Hospital from January 1, 2014, through June 30, 2014.	There was a random designation of babies into the intervention group (Family Connects Program) or control group (Treatment as usual). A nurse assessed and scored family intervention needs across 12 areas: parent and infant health, medical home, child care planning, parent- infant relationship, infant crying management, material support, family violence, maternal maltreatment history, maternal depression and anxiety, parental substance abuse, and social support. Based on this assessment, families received either a brief intervention, community resource connection, or emergency crisis support	The impact analysis encompassed 158 families in the intervention group and 158 in the control group. Nurses addressed minor problems in 52% of families and linked 42% to community resources. Child abuse investigations averaged 0.10 (0.30) in the intervention group versus 0.18 (0.56) in the control group (b = -0.09; 90%CI, -0.12 to -0.01; 95%CI, -0.18 to 0.01; P = .07). The intervention group had a maternal anxiety or depression rate of 18.2%, compared to 25.9% in the control group. (b = -7.70; 90%CI, -15.2 to -0.1; 95%CI, -16.6 to 1.3; P = .09). A nurse home visitation program for newborn families can be effectively implemented by a community agency with strong reach and quality.
Hans et al. (2018), Illinois [21]	To investigate the effectiveness of integrating community doulas into home-visiting programs to improve maternal and infant health outcomes	RCT: 312 young pregnant women with an average age of 18.4 years recruited from four communities in Illinois	Young pregnant women in high- poverty communities were put into control and intervention groups randomly. The intervention group received doula-home-visit services, whereas the control group received standard case management services. Community doulas provided support in childbirth education, breastfeeding, maternal health during pregnancy, and newborn care. Participants were interviewed during pregnancy as well as at 3 weeks and 3 months postpartum.	Mothers in the intervention group were significantly more likely to attend childbirth-preparation classes (50% vs. 10%; OR = 9.82, p < .01). No differences were observed between groups in rates of Caesarean delivery, birthweight, prematurity, or postpartum depression. Mothers in the intervention group also demonstrated safer infant care practices than mothers in the control group.
Horowitz et al. (2013), Boston [30]	To evaluate the effectiveness of the relationship (CARE) at improving the bond between mothers and their infants within the first nine months after birth.	RCT in three phases; 134 postpartum mother–infant pairs, representative of southeastern New England, United States, participated in the randomized controlled trial. 125 pairs were fully retained through the 9‑month protocol	Nurses coached depressed mothers to improve their interactions with their infants by addressing specific behaviors and encouraging more positive responses	Both the treatment and control groups showed notable improvements in mother-infant interaction quality and reductions in depression severity. No significant differences were observed between the treatment and control groups in maternal–infant relational effectiveness, caregiver responsiveness, or postpartum depression symptom severity
Latendresse et al. (2021), Utah [31]	To determine the effectiveness of telehealth intervention in preventing and treating perinatal depressive symptoms	Pilot, observational study, 47 participants (39 prenatal, 8 postpartum).	Weekly one-hour group videoconference sessions over 8 weeks, led by trained mental health professionals, integrating mindfulness- based cognitive therapy (MBCT), tailored to pregnant and postpartum women.	At baseline, 51.1% had mild to moderate perinatal depression (EPDS 10–20), while 48.9% were at high risk. Seventy percent attended ≥5 sessions. EPDS scores declined among those with current symptoms and remained stable among high-risk participants, suggesting preventive benefit. The study demonstrated that video conference intervention is a viable and effective method for delivery of care intended for reduction and prevention of perinatal depression symptoms.
Barrera et al.(2022) California, USA [32]	To determine the effectiveness of digital interventions in preventing and treating postpartum depression	Naturalistic, Digital Observational intervention; 208 participants (38 pregnant, 39 postpartum women; 131 health providers).	Eight online lessons adapted from the Mothers and Babies program, focused on CBT skills for mood management.	76% viewed at least one lesson, but only 4.4% completed all eight. Lesson 1 was most accessed (50.9%). Perinatal women engaged more with interactive content than providers (33.3% vs. 22.9%). Lessons were rated favorably, though feedback highlighted the need for tailoring and improved interactivity. The study concluded that the eMB course could be a promising digital intervention in addressing PPD
Danaher et al. (2013), Iowa [34]	To evaluate a web-based treatment for PPD's viability, acceptability, and possible effectiveness	A prospective pilot-based study; 53 postpartum women (<9 months postpartum, >18 years old with home internet connection and personal email use, EPDS score 12–20, or PHQ score 10–19)	Six-session interactive web-based program with structured CBT content, reminders, and guided activities.	87% completed all sessions; mean 20.1 visits, 5.1 hours total use. At pretest, 55% met PHQ-9 criteria for minor/major depression; by posttest, 90% of these no longer met criteria. Improvements were sustained at 6-month follow-up. The study found that participants who had previously fulfilled the PHQ-9 criteria for depression no longer did so at the 6-month follow-up, indicating increased use and efficacy of the web-based intervention
Baumel et al. (2018), New York [36]	To explore the integration of a digital peer-support platform into the treatment regimen for women diagnosed with PPD	Non- randomized design: 19 mothers diagnosed with PPD.	Online peer-support platform offering 24/7 emotional support from trained volunteers, guided by a study coach.	Participants engaged a median of 12 times (~175 minutes), mostly during evening/night hours. EPDS scores declined significantly (P < .001, Cohen d = 1.17). While differences vs. treatment-as-usual were not statistically significant, a medium effect size favored 7Cups (P = .05, Cohen d = 0.58)
McHale et al. (2022), Florida [37]	To examine the efficacy of prenatal intervention called FCC to improve healthy co- parenting dynamics and family environment among unmarried, low-income African American and mixed-race couples	RCT: 138 unmarried Black and mixed‑race mother–father dyads expecting their first child.	FCC, consisting of six prenatal dyadic sessions led by a male–female mentor team. Sessions emphasized communication, collaboration, and problem‑solving skills, delivered alongside treatment‑as‑usual (TAU).	Mothers in the intervention group reported more positive coparenting communication at three months postpartum compared to controls. Fathers in the intervention group spent significantly more time with their child, indicating enhanced paternal involvement. Both mothers and fathers in the intervention group reported significant reductions in psychological intimate partner violence (IPV). Both groups showed reductions in self‑reported depression over time, but no significant differences were observed between intervention and control groups.
Little et al. (2023), California [43]	To assess whether an effective intervention is necessary to lessen postpartum depression symptoms.	A randomized two-arm, parallel-group trial; 100 low-income mothers recruited between February 2018 and June 2019; 50 randomized to intervention and 50 to waitlist control.	During a prenatal home visit, the intervention group received an Ergo Baby Omni 360 carrier with usage instructions. Control mothers received the same carrier at 6 months postpartum. Recruitment was conducted by culturally matched Community Health Workers, and participants completed electronic surveys at late pregnancy and 6 weeks postpartum	Carrier usage was significantly higher in the intervention group (β = 2.69, SE = 0.347, p < .001, 95% CI = 2.08–3.41). At six weeks postpartum, mothers in the intervention group reported fewer depressive symptoms compared to controls (β = −0.541, p = .042). The intervention was well received, with 79.5% of mothers using the carrier in the first six weeks. No dose-dependent effect was observed within the intervention group.
South et al.(2021), *Pennsylvania* [46]	To examine the impact of spending time in nature on PPD.	Pilot RCT; 36 postpartum women from low-income, majority Black neighborhoods	A four-week program combining a Nature Coach, digital nudges, and personalized goal feedback. Nature visit frequency and duration were tracked via GPS.	Compared to controls, intervention participants showed a strong trend toward longer duration and higher frequency of nature visits (IRR 2.6, 95% CI 0.96–2.75, p = 0.059) Among women who completed the intervention (13 of 17), nature visits were three times higher than controls (IRR 3.1, 95% CI 1.16–3.14, p = 0.025). No significant differences were found in EPDS scores, likely limited by small sample size.

**Table 2:** Studies divided as per categorical themes and the contributing factors to postpartum depression they address.

Theme	Contributing factors addressed	Study
Theme 1: Home Visits	Limited Emotional Support, Access to community resources and barriers to Healthcare access, Poor Health Literacy, Low Maternal-Infant Interaction, Child maltreatment.	Study 1: Maternal health literacy progression among rural perinatal women [18]
		Study 2: Effect of a community agency-administered nurse home visitation program on program use and maternal and infant health outcomes [19]
		Study 3: Impact of Doula- Home-Visiting Services on Maternal and Infant Health [22]
		Study 4: Nurse home visits improve maternal/infant interaction and decrease severity of postpartum depression [30]
Theme 2: Telehealth, Online and Digital Interventions for Maternal Mental Health	Access to Care, Socioeconomic Barriers, Barriers to Traditional Mental Health Support	Study 5: A group videoconference intervention for reducing perinatal depressive symptoms [31]
		Study 6: Mothers and babies online course to prevent PPD [32]
		Study 7: A digital solution to reduce PPD symptoms [34]
		Study 8: Digital peer-support platform as an adjunct treatment for women with PPD [36]
Theme 3: Culturally Tailored Social Interventions for Underserved Families	Socioeconomic stress, Poor coparenting communication, Low father involvement, Psychological intimate partner violence (IPV), Cultural and systemic barriers	Study 9: Culturally grounded prenatal co-parenting Intervention for Unmarried Black parents [37]
Theme 4: Innovative Approaches to Maternal Mental Health and Bonding	Limited bonding opportunities, Lack of mental health support in low-income communities.	Study 10: An ergonomic infant carrier Intervention [43]
Theme 5: Nature-based Interventions for Maternal Mental Health	Mental Health, Lack of Greenspace Access	Study 11: Increasing time in greenspace among urban-dwelling postpartum women [46]

## Conclusions

This narrative review synthesized evidence across five categories of community‑based interventions to identify strategies with the greatest potential to reduce PPD and support maternal well‑being. Although the interventions varied widely in design, delivery, and target populations, several consistent patterns emerged. Guided digital CBT programs, particularly MMB, demonstrated the most robust and sustained reductions in PPD symptoms, highlighting the value of structured therapeutic content paired with human support. Synchronous telehealth mindfulness‑based groups also showed promising treatment and preventive effects, whereas self‑guided digital programs were limited by low engagement. Among home‑visiting models, the Family Connects program produced the clearest signal of improved maternal mental health, likely due to its comprehensive, biosocial approach that addresses both clinical needs and the social determinants that shape postpartum well‑being. In contrast, health‑literacy-focused nurse visits, community doula programs, and relationship‑focused coaching improved important proximal outcomes, such as parenting behaviors, coparenting communication, and reductions in psychological intimate partner violence, but did not consistently reduce PPD. Practical bonding tools, such as ergonomic infant carriers, and nature‑based interventions demonstrated feasibility and meaningful behavioral change, though larger trials are needed to determine their mental health impact.

Taken together, the evidence suggests that interventions are most effective when they combine evidence‑based mental health strategies with contextual supports that address relational, behavioral, and structural contributors to PPD. Guided digital CBT and comprehensive home‑visiting programs stand out as the most promising approaches, and integrating these models, such as embedding structured CBT within community‑based home‑visiting frameworks, may offer a more powerful and scalable strategy for reducing PPD, particularly in underserved populations. Future research should prioritize adequately powered trials, culturally responsive adaptation, and implementation strategies that enhance accessibility, engagement, and equity. By aligning intervention design with a biosocial understanding of PPD, community‑based programs can more effectively support maternal mental health and promote healthier early parenting experiences across diverse settings.

## References

[1] (2025). New York State Office of Mental Health. Postpartum Depression Screening Protocols and Tools: A Review of Evidence on Adequacy and Equity. Postpartum Depression Screening Protocols and Tools: A Review of Evidence on Adequacy and Equity, Chapter 384 of the Laws of 2022.

[2] O'Connor S, Su LJ (2023). Postpartum depressive symptoms: an analysis of social determinants using the pregnancy risk assessment monitoring system. Womens Health Rep (New Rochelle).

[3] World Health Organization. (2022 (2022). International Classification of Diseases 11th Revision (ICD‑ 11): 6E20 Mental or behavioural disorders associated with pregnancy, childbirth or the puerperium, without psychotic symptoms. https://icd.who.int/browse/2025-01/mms/en#1124422593.

[4] Zhao J, Zhang M (2024). Postpartum depression and risk factors among working women one year after delivery in Beijing, China: a cross-sectional study. Front Public Health.

[5] Ciolac L, Bernad ES, Tudor A (2025). Postpartum depression: interacting biological pathways and the promising validation of blood-based biomarkers. J Clin Med.

[6] (2025). Postpartum depression in New York City. NYC Vit Sign.

[7] Dennis CL, Dowswell T (2013). Psychosocial and psychological interventions for preventing postpartum depression. Cochrane Database Syst Rev.

[8] Maxwell D, Robinson SR, Rogers K (2019). "I keep it to myself": a qualitative meta-interpretive synthesis of experiences of postpartum depression among marginalised women. Health Soc Care Community.

[9] Byrnes L (2019). Perinatal mood and anxiety disorders: findings from focus groups of at risk women. Arch Psychiatr Nurs.

[10] Place JM, Renbarger K, Van De Griend K, Guinn M, Wheatley C, Holmes O (2024). Barriers to help-seeking for postpartum depression mapped onto the socio-ecological model and recommendations to address barriers. Front Glob Womens Health.

[11] (2007). Psychological and psychosocial interventions. Antenatal and Postnatal Mental Health: The NICE Guideline on Clinical Management and Service Guidance, No. 45.

[12] Hedman E, Ljótsson B, Lindefors N (2012). Cognitive behavior therapy via the Internet: a systematic review of applications, clinical efficacy and cost-effectiveness. Expert Rev Pharmacoecon Outcomes Res.

[13] Karyotaki E, Riper H, Twisk J (2017). Efficacy of self-guided internet-based cognitive behavioral therapy in the treatment of depressive symptoms: a meta-analysis of individual participant data. JAMA Psychiatry.

[14] Pugh NE, Hadjistavropoulos HD, Dirkse D (2016). A randomised controlled trial of therapist-assisted, internet-delivered cognitive behavior therapy for women with maternal depression. PLoS One.

[15] Sheeber LB, Seeley JR, Feil EG, Davis B, Sorensen E, Kosty DB, Lewinsohn PM (2012). Development and pilot evaluation of an Internet-facilitated cognitive-behavioral intervention for maternal depression. J Consult Clin Psychol.

[16] O'Mahen HA, Richards DA, Woodford J, Wilkinson E, McGinley J, Taylor RS, Warren FC (2014). Netmums: a phase II randomized controlled trial of a guided Internet behavioural activation treatment for postpartum depression. Psychol Med.

[17] Milgrom J, Danaher BG, Gemmill AW (2016). Internet cognitive behavioral therapy for women with postnatal depression: a randomized controlled trial of MumMoodBooster. J Med Internet Res.

[18] Mobley SC, Thomas SD, Sutherland DE, Hudgins J, Ange BL, Johnson MH (2014). Maternal health literacy progression among rural perinatal women. Matern Child Health J.

[19] Dodge KA, Goodman WB, Bai Y, O'Donnell K, Murphy RA (2019). Effect of a community agency-administered nurse home visitation program on program use and maternal and infant health outcomes: a randomized clinical trial. JAMA Netw Open.

[20] Baziyants GA, Dodge KA, Bai Y, Goodman WB, O'Donnell K, Murphy RA (2023). The effects of a universal short-term home visiting program: two-year impact on parenting behavior and parent mental health. Child Abuse Negl.

[21] Hans SL, Edwards RC, Zhang Y (2018). Randomized controlled trial of doula-home-visiting services: Impact on maternal and infant health. Matern Child Health J.

[22] Gruber KJ, Cupito SH, Dobson CF (2013). Impact of doulas on healthy birth outcomes. J Perinat Educ.

[23] Births and Beyond. (2021 (2026). Births and Beyond: Doula support. https://birthsandbeyond.com/studies-show.

[24] Abrams LS, Dornig K, Curran L (2009). Barriers to service use for postpartum depression symptoms among low-income ethnic minority mothers in the United States. Qual Health Res.

[25] Tandon SD, Parillo KM, Jenkins C, Duggan AK (2005). Formative evaluation of home visitors' role in addressing poor mental health, domestic violence, and substance abuse among low-income pregnant and parenting women. Matern Child Health J.

[26] Lancaster CA, Gold KJ, Flynn HA, Yoo H, Marcus SM, Davis MM (2010). Risk factors for depressive symptoms during pregnancy: a systematic review. Am J Obstet Gynecol.

[27] Lindensmith R (2018). Interventions to improve maternal-infant relationships in mothers with postpartum mood disorders. MCN Am J Matern Child Nurs.

[28] Sweet MA, Appelbaum MI (2004). Is home visiting an effective strategy? A meta-analytic review of home visiting programs for families with young children. Child Dev.

[29] Byatt N, Levin LL, Ziedonis D, Moore Simas TA, Allison J (2015). Enhancing participation in depression care in outpatient perinatal care settings: a systematic review. Obstet Gynecol.

[30] Horowitz JA, Murphy CA, Gregory K, Wojcik J, Pulcini J, Solon L (2013). Nurse home visits improve maternal/infant interaction and decrease severity of postpartum depression. J Obstet Gynecol Neonatal Nurs.

[31] Latendresse G, Bailey E, Iacob E, Murphy H, Pentecost R, Thompson N, Hogue C (2021). A group videoconference intervention for reducing perinatal depressive symptoms: a telehealth pilot study. J Midwifery Womens Health.

[32] Barrera AZ, Morris SY, Ruiz A (2022). Mothers and babies online course: participant characteristics and behaviors in a web-based prevention of postpartum depression intervention. Front Glob Womens Health.

[33] Gjerdingen D, Katon W, Rich DE (2008). Stepped care treatment of postpartum depression: a primary care-based management model. Womens Health Issues.

[34] Danaher BG, Milgrom J, Seeley JR (2013). MomMoodBooster web-based intervention for postpartum depression: feasibility trial results. J Med Internet Res.

[35] Alvidrez J, Azocar F (1999). Self-recognition of depression in public care women's clinic patients. J Womens Health Gend Based Med.

[36] Baumel A, Tinkelman A, Mathur N, Kane JM (2018). Digital peer-support platform (7Cups) as an adjunct treatment for women with postpartum depression: feasibility, acceptability, and preliminary efficacy study. JMIR Mhealth Uhealth.

[37] McHale JP, Stover CS, Dubé C, Sirotkin YS, Lewis S, McKay K (2022). A culturally grounded prenatal coparenting intervention: results of a randomized controlled trial with unmarried Black parents. J Fam Psychol.

[38] Baucom KJ, Chen XS, Perry NS, Revolorio KY, Reina A, Christensen A (2018). Recruitment and retention of low-SES ethnic minority couples in intervention research at the transition to parenthood. Fam Process.

[39] Karney BR, Kreitz MA, Sweeny KE, Ganong L (2004). Obstacles to ethnic diversity in marital research: on the failure of good intentions. J Soc Pers Relationship.

[40] Tandon SD, Leis JA, Mendelson T, Perry DF, Kemp K (2014). Six-month outcomes from a randomized controlled trial to prevent perinatal depression in low-income home visiting clients. Matern Child Health J.

[41] Cowan CP, Cowan PA, Pruett MK, Pruett K (2007). An approach to preventing coparenting conflict and divorce in low-income families: strengthening couple relationships and fostering fathers' involvement. Fam Process.

[42] Barlow A, Mullany B, Neault N (2013). Effect of a paraprofessional home-visiting intervention on American Indian teen mothers’ and infants’ behavioral risks: a randomized controlled trial. Am J Psychiatry.

[43] Little EE, Bain L, Hahn-Holbrook J (2023). Randomized controlled trial to prevent postpartum depressive symptomatology: an infant carrier intervention. J Affect Disord.

[44] Dagher RK, Bruckheim HE, Colpe LJ, Edwards E, White DB (2021). Perinatal depression: challenges and opportunities. J Womens Health (Larchmt).

[45] Howell EA, Mora PA, Horowitz CR, Leventhal H (2005). Racial and ethnic differences in factors associated with early postpartum depressive symptoms. Obstet Gynecol.

[46] South EC, Lee K, Oyekanmi K (2021). Nurtured in nature: a pilot randomized controlled trial to increase time in greenspace among urban-dwelling postpartum women. J Urban Health.

[47] Choe EY, Jorgensen A, Sheffield D (2020). Does a natural environment enhance the effectiveness of mindfulness-based stress reduction (MBSR)? Examining the mental health and wellbeing, and nature connectedness benefits. Landscape Urban Plan.

